# Seminal Plasma Anti-Müllerian Hormone: A Potential AI-Boar Fertility Biomarker?

**DOI:** 10.3390/biology9040078

**Published:** 2020-04-10

**Authors:** Isabel Barranco, Beatriz Fernandez-Fuertes, Lorena Padilla, Ariadna Delgado-Bermúdez, Asta Tvarijonaviciute, Marc Yeste

**Affiliations:** 1 Biotechnology of Animal and Human Reproduction (TechnoSperm), Institute of Food and Agricultural Technology, University of Girona, E-17003 Girona, Spain; beatriz.fernandez@udg.edu (B.F.-F.); ariadna.delgado@udg.edu (A.D.-B.); marc.yeste@udg.edu (M.Y.); 2 Unit of Cell Biology, Department of Biology, Faculty of Sciences, University of Girona, E-17003 Girona, Spain; 3 Department of Medicine and Animal Surgery, Faculty of Veterinary Medicine, University of Murcia, E-30100 Murcia, Spain; lorenaconcepcion.padilla@um.es (L.P.); asta@um.es (A.T.)

**Keywords:** Anti-Müllerian hormone, artificial insemination, fertility, inter-ejaculate variability, seminal plasma, porcine, sperm

## Abstract

The anti-Müllerian hormone (AMH), a Sertoli cell-secreted glycoprotein that is present in seminal plasma (SP), is considered as a marker of spermatogenesis in humans. This study aimed to evaluate the presence of this hormone in boar SP, together with its putative relationship with sperm quality, function, and in vivo fertility parameters in liquid-stored semen samples. The concentration of SP-AMH was assessed in 126 ejaculates from artificial insemination (AI)-boars (n = 92) while using a commercial Enzyme-Linked ImmunoSorbent Assay (ELISA) kit with monoclonal antibodies specific for *Sus scrofa* AMH (CEA228Po, Cloud-clone). Sperm quality (concentration, motility, viability, and acrosome damage) and functionality (membrane lipid disorder and intracellular H_2_O_2_ generation) were assessed in semen samples at 0 and 72 h of liquid-storage. In addition, fertility parameters from 3113 sows inseminated with the AI-boars were recorded in terms of farrowing rate, litter size, number of stillbirths per litter, and the duration of pregnancy over a 12-month period. The results revealed that the SP-AMH concentration varied widely among boar ejaculates, with no differences among breeds. Moreover, the SP-AMH concentration proved to be a good predictive biomarker for sperm concentration (*p* ˂ 0.05), but poor for other sperm quality, functionality, and in vivo fertility parameters of liquid-stored semen samples from AI-boars.

## 1. Introduction

Artificial insemination (AI) with liquid-stored semen is the most widely used reproductive biotechnology in the swine industry, playing a crucial role in genetic progress and increasing animal productivity [1]. Boars that are included in AI-programs are selected by their genetic merit and by the results that were obtained from conventional semen analysis (including sperm concentration, sperm motility, and morphology), as some of these sperm parameters correlate with in vivo fertility [2,3]. However, boars that have apparently good quality semen (based on these traditional tests) differ in the ability of their sperm to withstand liquid-storage, as well as in their field fertility [4,5,6]. It has been estimated that the proportion of sub-fertile boars in AI-centers ranges from 5 to 7% [1]. The inclusion of these boars in AI-programs, whose semen pass conventional quality checks but fails to achieve a high rate of pregnancies, leads to important economic losses at the farm level, as well as at the AI centers. Therefore, many efforts have been made in the last years to identify biomarkers of sperm function and fertility, particularly in seminal plasma (SP), in order to improve our ability to predict boar fertility. This fluid interacts with sperm at the time of ejaculation and subsequently with the female genital tract [7], making it a good candidate for containing biomarkers of fertility. Indeed, it has been recently demonstrated that the concentration of certain SP proteins, such as transforming growth factor-β1 (TGF-β1), is related to liquid-stored and cryopreserved sperm quality and function, as well as to in vivo boar fertility outcomes [8,9,10,11]. In humans [12,13,14,15], there is evidence for another member of the TGF-β-superfamily, the anti-Müllerian hormone (AMH), playing a role in male fertility. Thus, this protein could serve as a potential biomarker of boar fertility.

The AMH is a 140 kDa dimeric glycoprotein that is involved in the differentiation of the male reproductive tract [16]. Specifically, this hormone is expressed in Sertoli cells and it is responsible for the regression of the Müllerian ducts in the male fetus [16]. The AMH has been described and quantified in human SP [12,13,14,15,17,18,19,20]. The presence of this hormone in SP is of interest for the study of male reproduction as it has been suggested to provide information regarding the state of spermatogenesis [21]. In addition, the fact that SP-AMH is able to bind to the sperm surface suggests a role for this hormone on sperm function after ejaculation [22]. However, there is conflicting data in the literature regarding this point, since studies that have been performed in humans have demonstrated that SP-AMH concentration can be positively, negatively, or not related with sperm quantity and quality parameters [13,14,15,17,18,19]. Although the relationship between SP-AMH and fertility in humans is also controversial, most of the studies have revealed differences in SP-AMH concentration between fertile and infertile men, with the latter showing lower SP-AMH concentrations [12,13,14]. In contrast, to the best of our knowledge, there are currently no data available on the putative relationship between SP-AMH and sperm function and fertility in any livestock species, including the pig. Therefore, the aim of the present study was to evaluate the relationship between SP-AMH concentration and sperm quality, function, and in vivo fertility of boars that were included in AI-programs. 

## 2. Materials and Methods

### 2.1. Reagents and Media

The reagents that were used in the study were of analytical grade and, unless otherwise stated, acquired from Merck KGaA (Darmstadt, Germany). Fluorochromes were purchased from Molecular Probes (Thermo Fisher Scientific; Waltham, MA, USA).

### 2.2. Animals and Ejaculates

All of the procedures involving animals were performed according to European guidelines (Directive 2010-63-EU of the European Parliament and the Council of the European Union, 2010) and approved by the Bioethics Committee of Murcia University (research code: 639/2012).

Entire ejaculates (n = 126) were collected from 92 mature and fertile AI-boars from four different breeds (Landrace, Large White, Duroc and Pietrain) while using a semi-automatic collection method (Collectis^®^, IMV Technologies, L’Aigle, France). At the time of sample collection, the AI-boars were undergoing regular semen collection (twice per week) to produce commercial liquid-semen AI-doses (Calasparra, Murcia, Topigs-Norsvin, Spain). All of the ejaculates used in the present study satisfied the sperm quality requirements for the preparation of liquid semen AI-doses (˃70% motile spermatozoa and ˃75% of morphologically normal spermatozoa). In addition, all of the AI-boars included in the study were free of chromosomal translocations (sperm nuclear chromatin fragmentation rate ˂3%).

### 2.3. Seminal Plasma Processing and Storage

Right after ejaculate collection, the SP was separated from sperm by double centrifugation of the entire ejaculates (1500× *g* for 10 min. at room temperature (Rotofix 32A; Hettich Centrifuge UK, Newport Pagnell, Buckinghamshire, England, UK)). Subsequently, all of the SP samples were examined under a microscope (Eclipse E400; Nikon, Tokyo, Japan) to confirm the absence of sperm. Finally, the samples were aliquoted into three mL-cryotubes and stored at −80 °C (Ultra Low Freezer; Haier Inc., Qingdao, China) until the concentration of the AMH was measured.

### 2.4. Measurement of SP-AMH Concentration 

The concentration of the AMH in SP was assessed while using a commercially available competitive inhibition ELISA kit with monoclonal antibodies specific for porcine AMH (CEA228Po, Cloud-clone, BioNovacientifica S.L, Madrid, Spain) following the manufacturer’s instructions. Five standard points (ranging from 0.370–30 ng/mL) were added in duplicate to the plate to create the standard curve. All of the SP samples were thawed, diluted in PBS (1:80; v:v), and then added in duplicates to the plate along with their duplicate blank controls (standard diluent). Detection Reagent A was added to each well and the plates were incubated for 60 min at 37 °C in darkness. Following incubation, the contents of each well were aspirated and washed three times with washing buffer while using an automated plate-washer (ELx50/8RDS, Bio-Tek Instruments, Winooski, VT, USA). After blotting the plate with absorbent paper to remove any remaining washing buffer, Detection Reagent B was added to each well and then incubated for further 30 min. at 37 °C in darkness. The plate was then washed five times with washing buffer, and the substrate solution was added and incubated for 15 min. at 37 °C in darkness. During this incubation, a color change proportional to the amount of bound AMH takes place. Finally, stop solution was added to each well and, within 5 min., absorbance at 450 nm was measured using a micro-plate reader (PowerWave XS; Bio-Tek Instruments). Concentrations of AMH were expressed as ng/mL. The intra- and inter-assay coefficient variations were below 10%, exhibiting high linearity under serial dilutions.

### 2.5. Assessment of Sperm Quality and Functionality Parameters

A total of six different sperm quality and functionality parameters were assessed: (1) concentration, (2) motility (total and progressive), (3) viability, (4) acrosome damage, (5) membrane lipid disorder of viable sperm, and (6) basal production of intracellular H_2_O_2_ by viable sperm. 

Sperm concentration was objectively evaluated using an automated cell counter (NucleoCounter^®^ NC-100^TM^; ChemoMetec, Allerod, Denmark). Sperm motility was assessed while using a computer assisted sperm analyzer (CASA, ISASV1^®^, Proiser R+D S.L., Paterna, Spain). Briefly, 5 μL of extended semen (20−30 × 10^6^ sperm/mL in Biosem+ extender (Magapor, Zaragoza, Spain)) were placed in a pre-warmed (38 °C) Makler chamber (Sefi Medical Instruments, Haifa, Israel). Six to ten fields were acquired, so that ˃600 sperm were analyzed per semen sample. Sperm motility variables, such as the percentages of total motile (sperm with an average path velocity ≥20 μm/s) and progressively motile sperm (sperm with rapid and progressive movement with a straight-line velocity ≥40 μm/s), were recorded.

Sperm viability, acrosome damage, membrane lipid disorder, and H_2_O_2_ generation in viable sperm were evaluated by flow cytometry using a BD FACS Canto II flow cytometer (Becton Dickinson & Company, Franklin Lakes, NJ, USA). Prior to the addition of fluorochromes, each semen sample was diluted to a final concentration of 30 × 10^6^ sperm/mL in Biosem+. A total of three technical replicates (with a minimum of 1 × 10^4^ sperm events positive to Hoechst 33342 (H-42) dye) were assessed for each semen sample and sperm parameter.

In order to analyze sperm viability and acrosome damage, 100 μL of each extended semen sample were stained with 3 μL H-42 (0.05 mg/mL in phosphate buffered saline (BS: NaCl 139 mM, KCl 2.7 mM, KH_2_PO_4_ 1.5 mM, Na_2_HPO_4_·7H_2_O 8.1 mM; with 0.058 g/L penicillin G and 0.05g/L streptomycin sulphate; pH 7.1 ± 0.06; 289 ± 3 mOsmol/kg)), 2 μL propidium iodide (PI, 0.5 mg/mL in PBS), and 2 μL fluorescein-conjugated peanut agglutinin (PNA-FITC, 100 μg/mL in PBS) for 10 min. at 38 °C in darkness (Sanyo MIR-153 incubator, Gemini BV, Apeldoorn, Netherlands). The samples were then diluted in 400 μL PBS before flow cytometry analysis. Results are presented as the percentage of viable spermatozoa with an intact acrosome membrane (H-42^+^/PI^−^/PNA-FITC^-^).

To assess the membrane lipid disorder in viable sperm, 50 μL of each extended semen sample were stained with 2.5 μL H-42 (0.05 mg/mL in PBS), 10 μL Yo-Pro-1 (2.5 μM in dymetilsulfoxide [DMSO]) in 950 μL of PBS, and incubated for 8 min. at 38 °C in darkness. Before flow cytometry analysis, 26 μL of Merocyanine 540 (M-540, 0.1 mM in DMSO) was added to each sample, and then incubated for further 2 min. under the same conditions. The results are presented as the percentage of viable spermatozoa with high plasma membrane fluidity (H-42^+^/Yo-Pro-1^−^/M-540^+^).

Finally, the basal intracellular generation of H_2_O_2_ in viable spermatozoa was assessed following the procedure that was described by Guthrie and Welch [23], with slight modifications. Briefly, 50 μL of the semen samples were stained with 1.5 μL of H-42 (0.05 mg/mL in PBS), 1 μL of PI (0.5 mg/mL in PBS), and 1 μL of 5- and 6-chloromethyl-2′,7′-dichlorodihydrofluorescein diacetate acetyl ester (CM-H_2_DCFDA, 1 mM in DMSO) in 950 μL of PBS and incubated for 30 min. at 38 °C in darkness. As a control, a similar semen sample that was incubated with 1 μL of tert-butyl hydroperoxide solution (70% in distilled water) was used. The results are presented as the percentage of viable sperm with high intracellular H_2_O_2_ generation (H-42^+^/PI^−^/2′,7′-di-chlorofluorescein [DCF]^+^). 

### 2.6. Experimental Design

#### 2.6.1. Experiment 1: SP-AMH Concentration Variability Among Breeds 

For Experiment 1, a total of 49 entire ejaculates (one ejaculate per boar) were collected from boars (one ejaculate per boar) of different breeds: Landrace (n = 19), Large White (n = 9), Pietrain (n = 13), and Duroc (n = 8). The SP was obtained from each ejaculate and the AMH concentration was measured. 

#### 2.6.2. Experiment 2: Association Between SP-AMH Concentration and Sperm Quality and Functionality Parameters of Semen Samples Stored at 17 °C

In Experiment 2, a total of 26 ejaculates (one ejaculate per boar) were split into three aliquots: the first aliquot was used to assess sperm concentration; the second aliquot was used for recovery of SP, in which the AMH concentration was assessed; and, the third one was extended following the protocol for preparation of a commercial AI-dose (30 × 10^6^ sperm/mL in Biosem+), and sperm quality and functionality parameters (motility [total and progressive], viability, acrosome damage, membrane lipid disorder in viable spermatozoa, and basal production of intracellular H_2_O_2_ by viable spermatozoa) were assessed at 0 and 72 h of storage at 17 °C (FOC 120E Cooled Incubator; VELP Scientifica, Usmate, Italy).

#### 2.6.3. Experiment 3: Association Between SP-AMH Concentration and In Vivo Fertility Outcomes of Semen Samples Stored at 17 °C

In Experiment 3, a total of 51 ejaculates were collected from 17 AI-boars (three ejaculates per boar) during a 12-month period (one ejaculate/per boar/every four months). The SP was separated from sperm to measure AMH-concentration in this fluid. Liquid-stored semen AI-doses (2400 × 10^6^ of total spermatozoa in 80 mL) from these 17 AI-boars were used to cervically inseminate (2–3 times per estrus) a total of 3113 Landrace and Large White multiparous (1–7 prior litters) sows during this 12-month period. Each boar was used to inseminate at least 73 sows (ranging from 73 to 519 sows). Although the sows were housed in different farms across Spain, they were subjected to the same management conditions. The fertility parameters that were recorded over a 12-month period were: (1) farrowing rate (the proportion of inseminated sows that farrowed), (2) litter size (the total number of piglets born per litter), (3) the number of stillbirths per litter, and (4) the duration of pregnancy (measured in days). 

### 2.7. Statistical Analysis

The results were analyzed using IBM SPSS Statistics 25.0 (IBM Corp., Armonk, NY, USA). Shapiro–Wilk and Levene tests were used to check the assumption of normality and homogeneity of variances in the residual data for each parameter. In Experiment 1, one-way analysis of variance (ANOVA) followed by post-hoc Sidak test were used to evaluate the differences in SP-AMH concentration among ejaculates from different breeds. In Experiment 2, a two-step, hierarchical cluster analysis that was based on the log-likelihood distance and the Schwarz’s Bayesian Criterion was run to classify ejaculates into two groups (with high- or low- SP-AMH concentration). A repeated measures ANOVA was performed to evaluate the putative differences on sperm quality and functionality parameters between the two SP-AMH groups at the two evaluation storage times (0 and 72 h of liquid storage at 17 °C). Inter-subjects factor was the SP-AMH group (high- and low- SP-AMH concentration) and intra-subjects factor was the time of storage (0 h and 72 h); pair-wise comparisons were calculated using the Sidak test. In Experiment 3, to isolate and identify the direct effect of boar on each fertility parameter the in vivo fertility data were adjusted for parameters that were associated with the farm and sow by a multivariate statistical model [24]. In this experiment, a two-step, hierarchical cluster analysis that was based on the log-likelihood distance and the Schwarz’s Bayesian Criterion was also performed to identify the occurring groups of boars within the data of SP-AMH concentration; two groups were found (with high- or low- SP-AMH concentration). An independent *t*-test was performed to identify the differences on the in vivo fertility parameters between the two groups. The data are shown as mean ± standard error of the mean (SEM).

## 3. Results

### 3.1. Experiment 1: SP-AMH Concentration Variability Among Breeds

**Figure 1:**
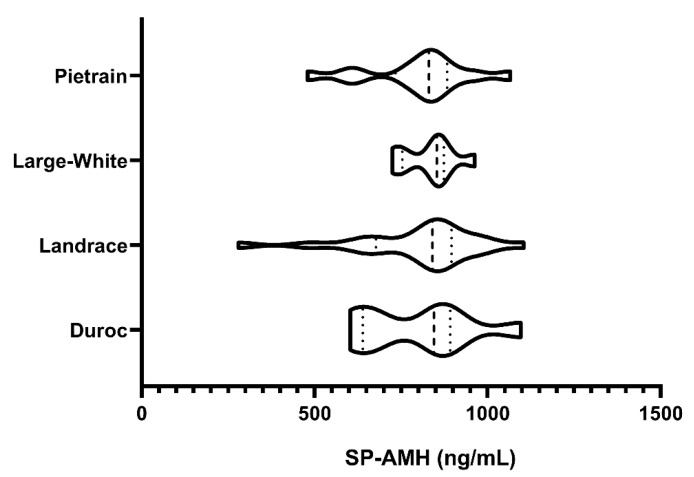
Violin plots representing concentration levels and distribution of the anti-Müllerian hormone (ng/mL) in seminal plasma (SP-AMH) from ejaculates (one per boar) of different pig breeds (Pietrain, n = 13; Large White, n = 9; Landrace, n = 19; and Duroc, n = 8). Dashed line represents the median and dotted lines the 25 and 75% quartiles. No significant differences in SP-AMH concentration were found between breeds.

Concentrations of SP-AMH differed widely among the 49 ejaculates, ranging from 279.83 to 1104.72 ng/mL. No differences between breeds were found (*p* > 0.05; Figure 1), as evidenced by similar SP-AMH levels in all studied breeds: Duroc (797.13 ± 52.93 ng/mL), Landrace (775.52 ± 42.37 ng/mL), Large White (832.21 ± 52.93 ng/mL), and Pietrain (801.27 ± 42.45 ng/mL).

### 3.2. Experiment 2: Association Between SP-AMH Concentration and Sperm Quality and Functionality Parameters of Semen Samples Stored at 17 °C

**Figure 2:**
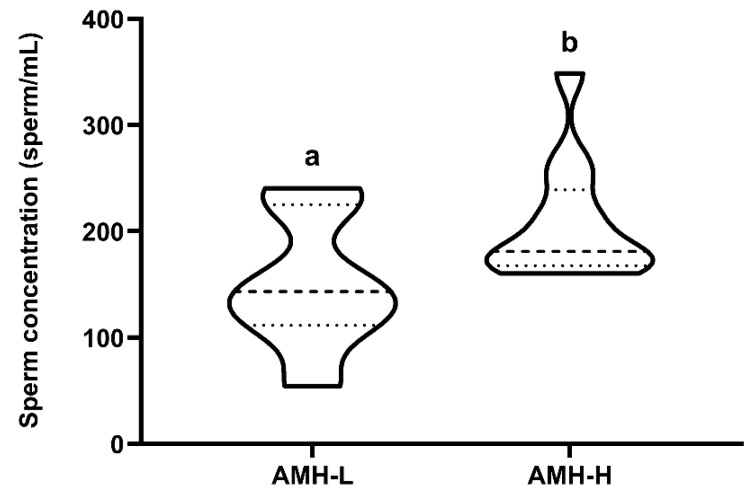
Relationship between concentration (ng/mL) of the anti-Müllerian hormone in boar seminal plasma (SP-AMH) and sperm concentration (sperm/mL). Violin plots represent levels and distribution of sperm concentration in boars hierarchically grouped (*p* ˂ 0.001) as having low- (AMH-L; ranging from 479.92 to 701.84 ng/mL, n = 7) or high- (AMH-H; ranging from 770.53 to 1096.72 ng/mL, n = 19) SP-AMH concentration. Dashed line represents the median and dotted lines the 25 and 75% quartiles. Different superscript letters (*a*–*b*) indicate significant differences between SP-AMH groups (*p* ˂ 0.05).

The 26 ejaculates were classified through hierarchical clustering (*p* < 0.001) into two groups: low (AMH-L; ranging from 479.92 to 701.84 ng/mL, n = 7) or high (AMH-H; ranging from 770.53 to 1096.72 ng/mL, n = 19) SP-AMH concentration. The ejaculates in the AMH-H group had higher sperm concentration than those in the AMH-L group (207.60 ± 16.22 sperm/mL vs. 149.85 ± 24.55 sperm/mL, respectively; *p* < 0.05; Figure 2). 

**Figure 3:**
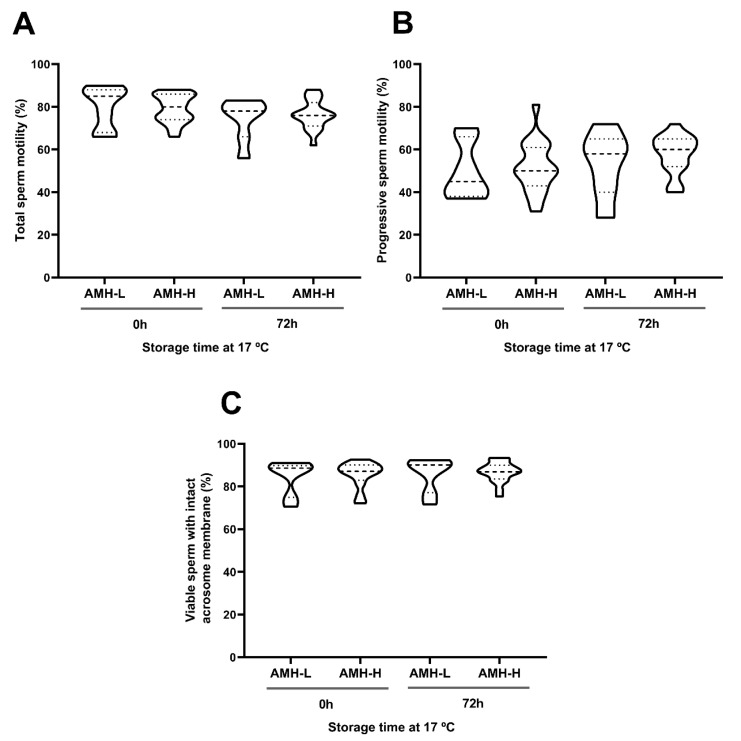
Association between the concentration of the anti-Müllerian hormone in boar seminal plasma (SP-AMH) and sperm quality parameters of semen samples stored at 17 °C up to 72 h. Violin plots represent percentages and distribution of (**A**) total motile sperm, (**B**) progressive motile sperm, and (**C**) viable sperm with intact acrosome membrane of semen samples (n = 26) assessed at 0 and 72 h after liquid-storage. The semen samples were hierarchically grouped (*p* ˂ 0.001) as presenting low- (AMH-L; ranging from 479.92 to 701.84 ng/mL, n = 7) or high- (AMH-H; ranging from 770.53 to 1096.72 ng/mL, n = 19) SP-AMH concentration. Dashed line represents the median and dotted lines the 25 and 75% quartiles. No significant differences were found between the SP-AMH groups in any sperm quality parameter.

**Figure 4:**
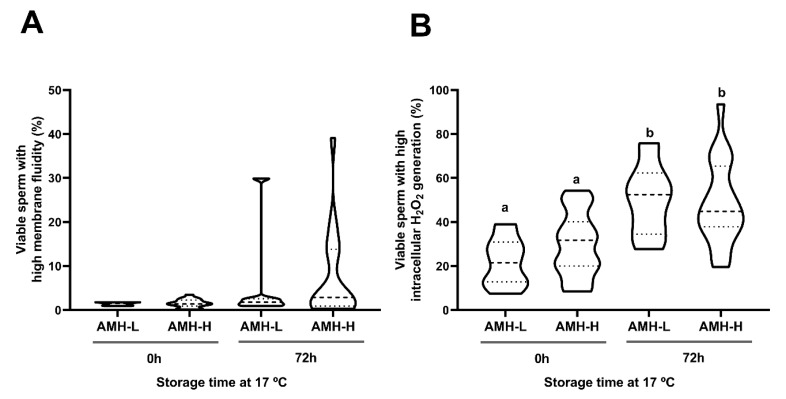
Association between the concentration of the anti-Müllerian hormone in boar seminal plasma (SP-AMH) and sperm functionality parameters of semen samples stored at 17 °C up to 72 h. Violin plots represent percentages and distribution of viable sperm with high (**A**) membrane fluidity and (**B**) intracellular H_2_O_2_ levels of semen samples (n = 26) assessed at 0 and 72 h of liquid-storage. The semen samples were hierarchically grouped (*p ˂* 0.001) as low- (AMH-L; ranging from 479.92 to 701.84 ng/mL, n = 7) or high- (AMH-H; ranging from 770.53 to 1096.72 ng/mL, n = 19) SP-AMH concentration. Dashed line represents the median and dotted lines the 25 and 75% quartiles. Different superscript letters (*a-b*) indicate significant differences between evaluation times within each SP-AMH group (*p ˂* 0.001).

Storage at 17 °C for 72 h had no effect on the motility, viability, acrosome damage or viable sperm with high plasma membrane fluidity. This was evidenced by comparable (*p* ˃ 0.05) percentages of total motile sperm at 0 and 72 h (AMH-H: 79.15 ± 1.52 and 76.42 ± 1.54, respectively; AMH-L: 80.14 ± 3.73 and 74.57 ± 3.77, respectively; Figure 3A), progressively motile sperm (AMH-H: 51.73 ± 2.73 and 57.84 ± 2.07, respectively; AMH-L: 49.85 ± 5.06 and 53.14 ± 5.75, respectively; Figure 3B), viable sperm with intact acrosome membrane (AMH-H: 85.58 ± 1.37 and 86.76 ± 1.09, respectively; AMH-L: 84.48 ± 3.09 and 85.78 ± 3.07, respectively; Figure 3C; Supplementary Table S1), and viable sperm with high membrane fluidity (AMH-H: 1.52 ± 0.21 and 7.37 ± 2.31, respectively; AMH-L: 1.42 ± 0.14 and 5.72 ± 4.03, respectively; Figure 4A). No differences between the AMH-H and AMH-L groups were observed in any of these parameters (*p* > 0.05).

As expected, liquid-storage had an effect on the intracellular H_2_O_2_ generation in sperm (*p* < 0.001). In both AMH groups, the percentages of viable sperm with high intracellular H_2_O_2_ levels increased from 0 to 72 h of liquid-storage (AMH-H: 29.75 ± 3.18 and 48.35 ± 4.26, respectively, *p* < 0.001; AMH-L: 21.72 ± 5.24 and 50.07 ± 7.02, respectively, *p* < 0.001; Figure 4B). However, no differences between the AMH-groups were observed at any time point (*p* > 0.05).

### 3.3. Experiment 3: Association Between SP-AMH Concentration and In Vivo Fertility Outcomes of Semen Samples Stored at 17 °C

**Figure 5:**
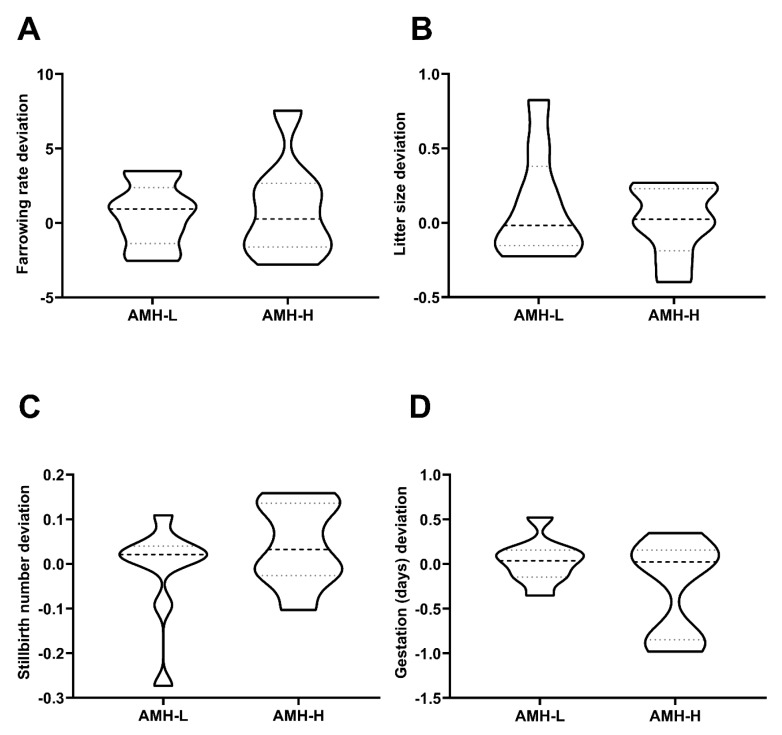
Association between the concentration of the anti-Müllerian hormone in seminal plasma (SP-AMH) and in vivo fertility parameters of boars in terms of direct boar effect. A total of 17 Artificial Insemination (AI)-boars (n = 3 ejaculates per boar) were classified as having low- (AMH-L; ranging from to 494.10 to 774.47 ng/mL, n = 9) or high- (AMH-H; ranging from 770.53 to 1096.72 ng/mL, n = 19) SP-AMH concentration in their ejaculates (mean of three ejaculates). Violin plots represent deviations and distribution of (**A**) farrowing rate deviation, (**B**) litter size deviation, (**C**) stillbirths number deviation, and (**D**) the gestation (days) deviation of these AI-boars. Dashed line represents the median and dotted lines the 25 and 75% quartiles. No significant differences were found between SP-AMH groups in any in vivo fertility parameter.

Similar to Experiment 2, the 17-AI boars that were used for the field fertility experiment were classified through hierarchical clustering (*p* < 0.001) into two groups according to their mean SP-AMH concentration (assessed in three ejaculates per boar). Again, the boars were grouped as displaying high (AMH-H: ranging from to 788.43 to 930.60 ng/mL; n = 8) or low (AMH-L: ranging from to 494.10 to 774.47 ng/mL; n = 9) SP-AMH concentration in their ejaculates. The SP-AMH concentration did not affect in vivo fertility outcomes (in terms of farrowing rate, litter size, number of stillbirths per litter, and duration of pregnancy; Figure 5). The farrowing rate deviations in the AMH-H and AMH-L boars were 0.89 ± 1.17 and 0.55 ± 0.71, respectively (*p* > 0.05; Figure 5A), whereas the litter size deviations were 0.01 ± 0.08 and 0.12 ± 0.11, respectively (Figure 5B). The number of stillbirths per litter deviation in AMH-H and AMH-L boars was 0.04 ± 0.03 and −0.01 ± 0.03, respectively (Figure 5C). Finally, the duration of pregnancy achieved by AMH-H boars deviated −0.25 ± 0.19, whereas those that were produced by AMH-L boars deviated 0.03 ± 0.08 (*p* > 0.05; Figure 5D). 

## 4. Discussion

To the best of our knowledge, this is the first report performed in a livestock species to assess the putative relationship between SP-AMH and sperm quality and the in vivo fertility of liquid-stored semen samples. In addition, this is also the first study in a mammalian species assessing the putative relationship between SP-AMH and sperm functionality parameters. Our results demonstrated that: (1) the AMH is present in boar SP, (2) a positive relationship exists between AMH concentration in SP and sperm concentration of boar ejaculates; however, (3) the abundance of the AMH in boar SP is not associated to differences in sperm quality, functionality, or to in vivo fertility of liquid-stored semen samples.

Using the AMH as a predictor of fertility has been widely researched from a female point of view. Circulating AMH concentrations have been shown to constitute a good biomarker of the ovarian reserve and antral follicle population in different mammalian species, including cattle, sheep, horse, pig, and humans [25,26,27,28,29]. However, in males, there is fewer evidence linking the AMH to fertility. Traditionally, the main purpose of the AMH was thought to revolve around the development of the male reproductive tract, inducing the regression of the Müllerian ducts in the fetus [16]. Indeed, the secretion of this hormone by Sertoli cells decreases after puberty [30]. However, AMH production does not completely cease during adult life. It is interesting to note that, after puberty, AMH secretion is higher at the apical pole of the Sertoli cell, towards the lumen of the seminiferous tubules, than basally, towards the interstitium and blood circulation [12,13,14,15,16,17,18,19,20,21,22,23,24,25,26,27,28,29,30]. As a result, higher concentrations of this hormone are found in SP in comparison to the serum of males [12,19,20]. This suggests a role of the AMH in spermatogenesis, while indicating that measuring SP-AMH, rather than serum-AMH, is more suitable when investigating the possible relationship between this hormone and sperm function. For this reason, in the present study, the link between AMH concentration in boar SP and sperm quality, functionality, and in vivo fertility was studied.

The results of this study revealed, for first time, that AMH is present in pig SP, showing average AMH concentration values that were higher than the AMH levels reported in human SP [12,15,18,20]. These results do not come as a surprise when one considers the differences existing in the concentration of other SP-hormones, such as testosterone, between humans and pigs [31,32]. Testosterone is thought to inhibit AMH transcription in the testis [21], likely through the negative regulation of NF-κB [33]. Similarly, studies that were performed in rodents reported that male mice that over-expressed AMH exhibited low testosterone levels [34], and intratesticular AMH injection in rats caused a decrease in the rate of testosterone synthesis [35], indicting a negative feedback between both hormones. Thus, the lower SP-testosterone concentration levels reported in boars [31] as compared with those found in men [32] could be the responsible for the higher SP-AMH levels found in boar SP samples.

The results of Experiment 1 evidenced a high variability in SP-AMH concentration among boars, which was not linked to their breed. Similarly, variation in SP-AMH between individuals has also been evidenced in humans [14,19,20,36]. Inter-boar variability in other SP-proteins, including other members of the TGFβ-superfamily, has also been reported [8,9,11], and might have a genetic origin [37]. Indeed, AMH gene polymorphisms condition circulating serum AMH levels in men [38], so it is likely that the AMH concentration in SP is also genetically determined.

In Experiment 2, the SP-AMH concentration was positively associated with sperm concentration. Our results are in agreement with studies that were carried out in humans [13,15,19], where a positive relationship between SP-AMH and sperm concentration was also observed. Considering that Sertoli cells are responsible for AMH secretion, and Sertoli cell number determines the number of sperm produced [39], this relationship was expected. Thus, higher levels of the AMH in SP could be indicative of a greater number of Sertoli cells, which could support a higher number of developing germ cells. Indeed, SP-AMH has been proposed as a biomarker of spermatogenesis in humans [15]. A link between AMH concentration and sperm quality is still subject to debate, despite its relationship to sperm concentration. In humans, some authors found that the SP-AMH concentration was positively related with some sperm quality parameters [13,14,19], whereas others reported a negative or no relationship between both [15,17,18]. In agreement with the study by Fujisawa et al. [15] and Nery et al. [18], the results obtained in the present study do not support a role for SP-AMH in sperm quality, as AMH concentration was not associated with differences in motility nor viability in samples stored at 17 °C for 72 h.

Aside from being present in SP, Fallat et al. [22] found the AMH to bind to the surface of the sperm head and, to a lesser extent, to the tail region. In addition, Hutson et al. [40] observed that the AMH inhibits protein tyrosine phosphorylation. Because protein tyrosine phosphorylation is required for acrosomal membrane exocytosis [41] and, together with evidence of AMH presence on the sperm head, the literature suggests a role for the AMH in preventing the sperm acrosome reaction. Accordingly, low SP-AMH concentration levels are related with a lack of intra-acrosomal enzymes in human sperm [20]. The acrosome status and membrane fluidity of boars with different levels of SP-AMH were assessed in Experiment 2 in order to shed light on the role played by SP-AMH on the pig sperm membrane. However, no relationship was found between these two sperm attributes and SP-AMH concentration levels. One caveat of the present study is that the semen samples were obtained from an AI-center, which means that all of the semen samples were of very high sperm quality. Thus, the high percentage of viable sperm with intact-acrosome and low membrane fluidity found in our samples might not provide enough power to detect small effects that are induced by the AMH. Further studies with a more diverse set of samples could aid in elucidating the role played by SP-AMH on sperm membrane status. Another parameter that was assessed in Experiment 2 was the levels of sperm intracellular H_2_O_2_. Recently, the concentration of a different TGFβ-superfamily member, TGFβ-3, in boar SP has been shown to be related to the ability of sperm to modulate intracellular H_2_O_2_ levels [11]. However, no differences were observed in sperm H_2_O_2_ levels between samples with high or low SP-AMH concentration, which would suggest that the AMH does not participate in the maintenance of intracellular H_2_O_2_ levels during storage at 17 °C. 

Finally, Experiment 3 aimed to determine the differences on in vivo fertility parameters between boars with different SP-AMH levels. Controversy also exists regarding the relationship between SP-AMH and fertility in humans. While some authors found differences in SP-AMH concentrations between fertile and infertile [12,13,14] patients, others did not observe that relationship [42]. The discrepancies between studies could be attributed to methodological causes, such as different criteria to select infertile men or the use of different methods to assess AMH concentration [43]. However, the presence of AMH receptors (such as AMH receptor II) in the human endometrium certainly points to the possibility of sperm-transported AMH to induce changes in the uterus [44]. This would not be the first instance of male-derived TGFβ-superfamily members inducing changes in the female reproductive environment. In mice, SP derived TGFβ elicits a cascade of immunological events that lead to maternal tolerance towards the semi-allogeneic embryo and improved implantation rates [45]. However, in the present study, the SP-AMH concentration did not affect the in vivo fertility outcomes of AI-boars. Again, all of the boars used in the study had very good fertility, which could mask any subtle effects that SP-AMH might have on fertility. Therefore, further studies in other species using a population with higher variation in fertility rates (such as the ones that are used for human studies) could provide better information in this regard.

## 5. Conclusions

In summary, the present study demonstrates the presence and variability of SP-AMH concentration among boar ejaculates, together with the positive relationship between AMH levels and sperm concentration. Therefore, the SP-AMH concentration proved to be a good predictive biomarker of sperm concentration, but poor for other sperm quality, functionality, and in vivo fertility parameters of liquid-stored semen samples from AI-boars.

## Supplementary Materials

The following are available online at https://www.mdpi.com/2079-7737/9/4/78/s1. Table S1: Data of sperm viability and acrosome damage assessed in boar semen samples (n = 26) at 0 and 72 h after liquid-storage at 17 °C. For flow cytometric analysis semen samples were stained with Hoechst 33342 (H-42), propidium iodide (PI) and fluorescein-conjugated peanut agglutinin (PNA-FITC). Semen samples were hierarchically grouped (*p* ˂ 0.001) as presenting low- (AMH-L; ranging from 479.92 to 701.84 ng/mL, n = 7) or high- (AMH-H; ranging from 770.53 to 1096.72 ng/mL, n = 19) seminal plasma (SP) anti-Müllerian hormone (AMH) concentration. Results are presented as the percentage (mean ± SEM) of (1) viable spermatozoa with an intact- (H-42+/PI-/PNA-FITC-) and non-intact-acrosome membrane (H-42+/PI-/PNA-FITC+) and (2) non-viable spermatozoa with an intact- (H-42+/PI+/PNA-FITC-), and non-intact- acrosome membrane (H-42+/PI+/PNA-FITC+) in each SP-AMH group.
