# Supplementary material for: Seminal Plasma Anti-Müllerian Hormone: A Potential AI-Boar Fertility Biomarker?
Source: Biology (Basel). 2020 Apr 10;9(4):78. doi: 10.3390/biology9040078 (PMC7236007; doi:10.3390/biology9040078)
Supplement: Supplementary file 1 [file biology-09-00078-s001.pdf]

SP-AMH group	Evaluation time-points	Viable sperm with intact acrosome membrane (%)	Viable sperm with non-intact acrosome membrane (%)	Non-viable sperm with intact acrosome membrane (%)	Non-viable sperm with non-intact acrosome membrane (%)
Low	0	84.48 ± 3.09	1.5 ± 0.16	2.87 ± 1.24	11.11 ± 2.09
High	0	85.58 ± 1.37	1.6 ± 0.25	2.89 ± 0.58	9.92 ± 1.03
Low	72	85.78 ± 3.07	2.32 ± 0.52	2.22 ± 0.78	9.67 ± 1.92
High	72	86.76 ± 1.09	2.54 ± 0.37	1.96 ± 0.41	8.73 ± 0.87
